# Subjective Well-Being in Healthcare Professionals in Colombia: On the Constitution of Subjectivity and the Ethics of Care in Times of the COVID-19 Pandemic

**DOI:** 10.3389/fpsyg.2021.773173

**Published:** 2021-11-11

**Authors:** Diego Fernando Barragán-Giraldo, Giovanni Anzola-Pardo, Maura Andrea Guerrero-Lucero

**Affiliations:** ^1^School of Humanities and Social Studies, Universidad de La Salle, Bogotá, Colombia; ^2^Directorate of International Relations, Universidad Externado de Colombia, Bogotá, Colombia; ^3^Faculty of Education Sciences, Universidad de La Salle, Bogotá, Colombia; ^4^Extension Office, Universidad Mariana, Pasto, Colombia

**Keywords:** subjective well-being, healthcare professionals (HCPs), subjectivity (European Education Thesaurus), ethics of care of the self, COVID-19, higher education, remote education of emergency

## Abstract

The purpose of this work is to reveal how subjective well-being has been generated in a group of professionals in the healthcare field in Colombia, who carried out postgraduate studies at the time of the pandemic caused by the novel SARS-CoV-2 coronavirus in a synchronous and remote learning course facilitated by employing digital technologies. Two methods were assumed, one was qualitative, taking into account some elements of narrative research and discourse analysis, and the other was quantitative, through a rapid reconnaissance survey. The research assumes the constitution of subjectivity from memory and everyday life, as well as the ethics of care concerning caring for oneself and others, as categories that were (re)signified with the narratives—and as notions that make up a theoretical corpus—to understand subjective well-being.

## Introduction

The COVID-19 outbreak disrupted traditional practices in higher education, pedagogy, and interpersonal relationships in the classroom, to the point of (re)configuring ways of managing education. From this perspective, various practical initiatives have sought to address this situation, seeking to generate, through micro-curricular adjustments, didactics, evaluative processes, and situations that allow better learning to establish subjective well-being in students. This has led to a rethinking of how the practices of different educational actors are associated, creating other pathways of social interaction in which digital technologies are put into play and, especially, the possibilities of remote education assisted from various technological developments.

Educational experiences that were previously developed in person had to be modified during the COVID-19 pandemic. Learning settings had to be adjusted to the needs of the pandemic situation to include other ways of accessing training processes. Therefore, faculty's practices were affected, as well as the didactic and pedagogical relationships that hitherto made possible the student well-being. Given that such adjustments implied assimilating digital skills to manage school relationships and making changes in practices that constitute subjective well-being, school inequalities related to access and the connectivity of individuals and communities inevitably emerged.

It is estimated that the pandemic caused by the novel SARS-CoV-2 coronavirus has had catastrophic effects, not only in terms of physical health and mortality but also in areas of mental health and the world economy, with social, political, and cultural consequences that are difficult to calculate (Levy, 2020). It is difficult to examine or compare how the new social and educational configurations are generated—or will be generated—and to foresee, from an almost antagonistic perspective, the generation of new knowledge from “new interactions” (Anzola-Pardo, 2019), especially in classrooms.

Higher education institutions (HEIs) face dualities with the progressive return to normality (gradual, permanent, or without people on campuses). According to UNESCO-IESALC (2020) as of May 2020, university closures had already affected approximately 23.4 million students in the world. To tackle this issue, the same organization launched the COVID-19 plan toward the reopening of higher education in Latin America and the Caribbean. The initiative indicates how the situation of HEIs affects students, identifying different States, where the majority of institutions are still on hold regarding openings. In the report, Nicaragua is noted as the only nation in the region with a policy of total opening. Taking a different approach, Colombia—with a fully vaccinated population of 28.45% as of August 2021—has opted for a partial (hybrid) opening.

The postponement of face-to-face activities and uncertainty limit a concrete resolution in the face of training processes since in most cases flexibility has become an essential aspect of continuity; however, spontaneity by adaptation prevails. Thus, faculty members were forced to transform face-to-face to virtual teaching—facing these situations without being fully prepared in the use of digital platforms, teaching, learning, and evaluation while maintaining, in some way, curricular proposals and their teaching techniques. Nevertheless, these new academic interactions make up educational modalities that, although rarely experienced in the past, generate positive pedagogical disruptions and different perspectives of mediation from frustration and being overwhelmed by adaptation through and to digital technologies.

Pedagogical settings should also be considered as a place of care in which actions of recognition of otherness are promoted. In educational and pedagogical practices, this takes place when the difficult becomes easy and learning experiences are stimulated. The relationship between faculty and the student becomes the site of human interactions to generate links and, therefore, learning propitiation. In this sense, subjective well-being is not something added but is rather a constitutive part of educational activity.

In the present study, subjective well-being was promoted in postgraduate students within the framework of the emergency remote education practices caused by the COVID-19 pandemic. The promotion of subjective well-being in higher education involves concrete actions on pedagogy in practice. The pedagogical actions are of a practical order; for this reason, they go beyond theory and are inscribed in the transformation that can be generated by those who participate. With the pandemic, the place of the experience has changed, leading faculty members to reconfigure ways of conceiving their educational relationships; thus, institutions and people have had to reinvent themselves.

The centrality of the pedagogical action used in this study is framed by the conception that practical knowledge (Phronesis) has to address a reflective notion before, during, and after action (Kemmis, 1986, 2009; Carr, 1995, 2004, 2005; Flyvbjerg, 2001; Kemmis et al., 2014; Barragán, 2015); in this case, in postgraduate training settings.

## Theoretical-Categorical Horizon

When speaking of the configuration of the mental well-being of human beings, the psychological is an important dimension in terms of the acceptance of personal development. This brings together the subjective, which recognizes the elements of satisfaction of one's configuration, and the social, in which configurations are sought in relationships with others (Joshanloo et al., 2018; Chung and Hahn, 2020). This research focuses on the subjective dimension from the perspective of well-being exclusively.

We assume subjective well-being as those practices that allow people to acquire a state of well-being with themselves and with others, in such a way that the configuration with the psychic and social being occurs. There are different traditions regarding this notion; however, it is relevant to understand that multiple inquiries about subjective well-being have incorporated affective indicators of happiness (Hedonic well-being), as well as cognitive evaluations of satisfaction before life. Similarly, elements related to personal growth, human development, and levels of self-acceptance have been considered, as well as the meaning of well-being, with the search for happiness and its relationship with the fulfillment of human beings (Keyes et al., 2002).

In general, two traditions have been established for the study of subjective well-being: one that emphasizes happiness (hedonic well-being) and one that deals with human potential (eudaimonic well-being) (Waterman, 1993; Ryan and Deci, 2001). In this framework of reference, various investigations explore subjective well-being in a less instrumental key, to the point that the Aristotelian tradition of understanding ethics is retaken (Deci and Ryan, 2008), especially in the search for eudaimonia, in its philosophical dimension, a fundamental issue in ethics (Waterman, 1990; Díaz et al., 2015). The search for eudaimonia in others generates well-being, beyond a hedonic satisfaction of individual needs (Huta et al., 2012). Joshanloo et al. (2018) insist on the need to explore eudaimonic well-being, which seeks to improve positive mental health through a rich understanding of psychological and social well-being, in a complementary relationship. It is about understanding that eudaimonic activities are foundational elements of subjective well-being (Martela and Sheldon, 2019). Hence, this type of well-being is sought in labor relations or other spheres of human relations (Russell, 2008; Chung and Hahn, 2020).

This inquiry makes an approximation of subjective well-being by exploring some dimensions from ethics. Thus, the categorical horizon of work encompasses the constitution of subjectivity and ethics of care, categories that operate as elements of methodological articulation, and the theoretical resignification of subjective well-being.

### Constitution of Subjectivity

The configuration of the notion of the subject has had a long journey. In Europe, Plato, Aristotle, and Seneca, and in China, Confucius—to name just a few thinkers—have addressed the question of the good life or the conditions that allow states of well-being. Descartes' concept of cogito discusses representation and places the subject as an instrumental archetype of understanding the human, emphasizing rationality as an all-encompassing notion of all spheres of understanding about the human and especially well-being. Known as “modern rationality” this condition has become an established hegemony in the interpretation of the human. This hegemonic approach has been widely criticized, as it constrains other approaches to the human (Gadamer, 1975; Frank, 1995; ŽiŽek, 1999; Santos, 2010; Barragán, 2012). Criticism of and resistance to this totalitarian approach to the notion of the subject frame the human as a network of interactions that are configured and, consequently, surpass the epistemological scientism that usually rules in different disciplines. In this context, the notion of the constitution of subjectivity appears as a construct that allows us to move beyond the restricted perspective of modern rationality. Constituting subjectivity means, in short, understanding that human beings have a system of relationships, which goes beyond the objectification of human activity. This theoretical construct emerges as opposed to the modern understanding of the subject concerning human action.

Two categories are relevant for understanding the constitution of subjectivity. The first is “memory,” which does not refer exclusively to the memories that can be brought to the present. Memory, as a possibility of establishing historicity, leads the human being to recognize themself. It is someone who recognizes the self in their context. It is about the Heideggerian Dasein which is, ultimately, the human being who is assumed to be situated in a “concrete there” (Heidegger, 2003). Memory—as a present loaded with the past and with the tension of the future—allows the individual to situate the self in their biography and is assumed to be situated in historical consciousness (Gadamer, 1975, 1986, 2002).

The second category for constituting subjectivity is “the everyday.” The human being, when moving in routine life, runs the risk of falling into the dark heaviness of everyday life (Heidegger, 2003). Everyday things and experiences can become irrelevant as they can become an unreflective routine that loses its value. When viewed with new eyes, the everyday is, par excellence, important to generate belonging in one's historicity (Gadamer, 1986; Ricoeur, 1990, 2010; Heidegger, 2003). The everyday speaks of the world: the one to which we have access daily and which is a motive for reflection.

### Ethics of Care

Caring for oneself and others, in terms of the pursuit of good and happiness, is the center of ethics. Aristóteles (1985, 2004) emphasizes the relationship between good (Agathon) and happiness (the Eudaimonic), as a possibility of ontological ethics. This approach to ethics goes beyond the deontological instrumentalization of moral action and values to focus on practices. Caring enables a human being to cope with existence; that is to say, with oneself. In this sense, the existential, particularity that which characterizes human actions—is always about the world and the experiences that derive from it.

Care also invites us to browse the world (Heidegger, 2003) and perceive existence from other perspectives and thus, explore other possibilities for configuring subjectivity. It implies, at the same time, questioning the surrounding world and one's existence. To exist is to take care of oneself (Gadamer, 1986; Ricoeur, 1990; Barragán, 2015).

It is in this questioning of existence and the world, that human beings take care of themselves and by extension others, as well as the material world: this always concerns the everyday. An ethics of care will have, as its axis and center, the search for good from the perspective of happiness and consequently, it implies assuming a caring attitude toward oneself and others. In this way, caring for oneself and caring for others are fundamental dimensions in the generation of subjective well-being.

## Materials and Methods

Two approaches were used in this work. The first was quantitative, using a rapid reconnaissance survey (Butler, 1995). The other was qualitative, as part of which experience was central to the methodological approach (Creswell, 1997; Creswell and Miller, 2000; Flyvbjerg, 2001). Experience is understood as a set of actions that refer to the meanings of life that individuals seek in specific situations concerning their practical knowledge, which are configured by entering into relationships with others (Barberousse, 1999; Flyvbjerg, 2001; Eikeland, 2008; Jay, 2009; Perreau, 2010; Grondin, 2014, 2018). For the methodological design, the main aspects of narrative research were taken into account, in which the experience gains value, insofar as it is related, to raise awareness about what happened and address future action patterns in the face of phenomena with equivalent characteristics (Delgado and Gutiérrez, 1999; Bolívar et al., 2001).

Within this framework, and considering that it is necessary to generate subjective well-being in higher education (Botha et al., 2019), the current work examines how subjective well-being has been generated in a group of professionals who work in the field of healthcare in Colombia. The group was carrying out postgraduate studies during the new SARS-CoV-2 coronavirus pandemic, in a synchronous remote learning course via digital technologies during the years 2020 and 2021.

The experience of professionals working in healthcare in seven Colombian capital cities (Bogotá, Neiva, Cúcuta, Armenia, Cartagena, Barranquilla, and Pasto) was documented. Participants carried out didactics to promote subjective well-being. For the present work, the following actions were taken into account: an activity called work with valuable objects; the production of testimonial narratives (TN), and, finally, a rapid reconnaissance survey (Butler, 1995), as seen in Table 1.

**Table 1:** Rapid reconnaissance survey—recurrence of words during courses.

**Considered questions**	**Yes**	**No**	**Categories**	**Word recurrence**	**Count**
Valuable objects as generators of well-being	98.78%	1.22%	Constitution of subjectivity	Life	32
				Meet	23
				Person	26
				Being	26
				Things	23
				Professional	22
				Object	17
Knowing classmates better generating well-being in the relationships	98.17%	1.83%	Ethics of care	Valuable	25
				Values	22
				Knowledge	21
				Ethics	18
				Wellness	13
				Life	12
				Colleagues	18
				Ours	17
				Other	14
Relevance of valuable objects in pedagogical terms	98.78%	1.22%	Pedagogical aspects	Class	54
				Allowed	32
				Appreciation	37
				Excellent	21
				Teacher	21
				Dynamic	17
				Knowledge	17
				Learning	10
				Tools	10

The first didactic activity served as a narrative trigger. It consisted of a work called “discussions around valuable objects,” where each participant put in an online application, a photograph of an object that was important to them, to promote moral practices and the moral culture of the group (Puig-Rovira, 2003; Puig-Rovira et al., 2012). Subsequently, each participant commented on the objects in writing and then, voluntarily, each one spoke about their images or asked classmates. The purpose of this activity was to generate the interaction of the group and that which they can recognize in others, through objects, human beings with similar feelings and characteristics.

The second activity was the preparation, by each participant, of a testimonial story in which they narrated situations that triggered some tension from ethics or morals in their professional practice in the field of healthcare during the COVID-19 pandemic. These narratives operated as an environment for narrative empowerment for the production of subjective well-being and, at the same time, as an information-gathering strategy.

Testimonial narratives, in their epistemology, are related to stories and life narratives. In both approaches “Life story” and “Life narrative,” a storytelling experience is presented that is open to interpretation that, by anchoring itself to the biography of the narrator, makes it possible to address the lived experience (Chanfrault-Duchet, 1987; Pujadas, 1992; Santamarina and Marinas, 1993; De Miguel, 1996; Atkinson, 1998; Bertaux and Kohli, 2003). However, TN are a type of abbreviated narrative that marks a person's biography and that, due to their relevance, become a reason for reflection for themselves and others. Relating generates the possibility of looking again at relevant events in people's lives, to open up the possibilities of exploring new pathways that allow us to face similar events.

A rapid reconnaissance survey was sent to the people who made the TN. It used yes/no questions and open responses, examining the experience of the participants in the two activities previously mentioned, and concerning the promotion of subjective well-being. It was also investigated relevant aspects of the academic space of remote learning mediated by digital technologies. The purpose of this strategy is to quickly approach a phenomenon and look for relevant aspects that can give general clues about the situation under investigation (Butler, 1995).

A questionnaire was (Goetz and LeCompte, 1988) sent to 179 students from two specializations, and who took the course in healthcare ethics during the years 2020 and 2021. The questionnaire was completed by 164 participants (91.62%), which corresponds to a 99% confidence level and a 3% margin of error.

Concurrent Triangulation (CT) was then applied to the themes derived from the qualitative and quantitative data gathered. The collection and analysis of data were conducted separately yet concurrently. The findings were integrated during the interpretation stage of the study, where equal priority was given to both types of data (Terrel, 2012). Onwuegbuzie and Leech (2006) explain that research design will be concurrent if the quantitative phase of the study does not inform or drive the qualitative phase, and vice versa. In this sense, the triangulation of information collected is based on what appeared in the evaluative discussions at work with valuable objects, the TN, and the reconnaissance survey (S).

A simple emergent coding was carried out, which was analyzed following the guidelines of content analysis (Krippendorf, 1990; Navarro and Díaz, 1999; Monge-Acuña, 2015) and the discursive emergence of narrative research that allows us to reflect upon life and also find the meta-discursive regularities regarding the categorical horizon established as an interpretive basis (Orofiamma, 2002; De Ryckel and Delvigne, 2010).

The population included 164 healthcare professionals in the fields of: Nursing, 63; medicine, 31; dentistry, 22; bacteriology, 9; physiotherapy, 7; psychology, 5; administration, 7; optometry, 5; epidemiology, 2; surgical instrumentation, 6; law, 4; economy, 3. Of these, 142 declared themselves to be female, 22 male, and 1 person preferred not to mention gender. Informed consent was requested from all participants and the data was guaranteed to be processed anonymously.

## Discussion

As mentioned in the materials and methods section, a rapid reconnaissance survey (Butler, 1995) allowed us to measure the perception of the activities carried out at the time of the research process. An analysis of the frequency of words led to our focus on the narratives based on the categories of the constitution of subjectivity and ethics of care. It should be mentioned that the group of healthcare professionals recognized the relevance of the pedagogical action in promoting subjective well-being, and 98.78% considered that the activity carried out during a class in the ethics and values of healthcare, with valuable objects, led them to greater knowledge as human beings, generating some sort of well-being. Only 1.22% did not perceive it that way.

In total, 98.17% considered that the activity on valuable objects led them to get to know their colleagues better, generating well-being in relationships, but 1.83% did not consider it this way. It is noteworthy that 99.39% of those who participated in the course recognized that these activities presented an opportunity to generate personal well-being and self-care during class, helping them to cope with remote learning mediated by virtual platforms in times of the COVID-19 pandemic; 0.61% did not consider it that way. These quantitative results made it possible to ratify the importance of generating subjective well-being from a pedagogical perspective in higher education and created the possibility of reviewing the phenomenon using the narratives that appear in the testimonies.

In the qualitative phase, examination of the narratives allowed us to capture different ways of understanding subjective well-being. It also enabled us to consider the constitution of participants' subjectivity and the ethics of care. In connection with the objective of the work, some reports are presented to allow us to further examine how subjective well-being was perceived by the professionals who participated in the research. This allows us to trace future pathways so as not to repeat past mistakes; and thus, narrating becomes an opportunity to recognize historicity (Gadamer, 1975) in a discursive key: “This experience taught us that the word has power when used assertively” (Physician 22, TN). Thus, memory, as an ethical trigger, promotes historical awareness training guidelines for healthcare professionals, as discussed in some reports:

“The principles and values instilled in our training make us look, from the perspective of ethics and morals, between what should be done and what it is done; each human being makes a decision, depending on his/her culture, values, and even convenience” (Nurse 13, TN).

“It reminded me of people and the value that each one has; in addition to the most important places in my life” (Nurse 21, S).

“It was a different activity, which kept us focused, and made us reflect on many areas of our lives, and made us remember the value of many things that surround us” (Odontologist 9, S).

“Our values and principles are not negotiable, although sometimes, without agreeing, we must respect the position and the way of thinking of people, avoiding judgments” (Odontologist 18, TN).

The narrative represents a situated knowledge that permeates the experience itself in terms of ways of subjecting themselves to this group of people, based on the decisions they have made, as outlined in this testimony: “I will remind myself regarding how important the decisions I have made are” (Nurse 42, S). Another participant observed that “There remains a great experience, which is prudence, as an act of knowing how to decide” (Doctor 18, TN).

Narrating what happened leads to the recognition that “remembering the values and different paradigms of society make us think about what it is good and acting well always brings us benefits and inner peace” (Doctor 17, S). The teleological sense of acting assumes dimensions of intrapersonal recognition: “we can become aware of the importance of being better human beings. Day after day, without having to trample anyone, and with the joy of knowing that what is done correctly is based on our principles” (Doctor 32, TN).

These narratives allow us to glimpse the ethical possibilities of acting, as practical knowledge (Aristóteles, 1985; Ricoeur, 1990; Gadamer, 2002), to build possible worlds from memory and the ethical question about what happened and what should be:

“A deathly silence, in a few seconds that became eternal; that moment so short and with a fine line that limits life and death, when you sedate the patient, pass the orotracheal tube, and see the patient's clinical response, it becomes a daily routine; many patients with happy satisfaction respond to the proposed therapy; many others do not, and only in sedation, their lack of oxygen leads them to respiratory failure and, unintentionally, that crucial moment arrives in which they decide to resuscitate a patient, having full knowledge that if you do so, the risk of infecting the whole team and yourself is everything! […]. In any society that aspires to humanistic healthcare practice, the work of a doctor, in particular, is related to preparation and motivation to offer help, without considering the difficulties that may arise, the obstacles that may have to be overcome, even going so far as to put one's life at risk, for the sake of saving the patient's life; however, how ethical is it to go?” (Doctor 2, TN).

Another relevant aspect is that narrating allowed participants to approach the everyday, as a reflective opportunity concerning what is valued by people. As one participant observed, “knowing what is valuable to someone, undoubtedly offers a lot of information, not only about the object itself, but that tells a story that allows us to intuit other things about people” (Bacteriologist 6, S). In this way, the experiences of others are valued, acting as a way to deepen views on oneself and the profession:

“It has helped me to put myself in other people's shoes; how they have lost their beloved ones, because of a virus and the big suffering when you are not able to say the last goodbye. As a nurse, I have set myself a new challenge in which I am willing to fight and support others who are in the fight against diseases that may not have a possible cure, treatment, or perhaps COVID-19—that we still do not know treatment to eliminate or cure. Seeing people die every day is very shocking to me. I think that the experience can help healthcare professionals, who at this moment feel demotivated by the pandemic situation, to be more human and to live a new story every day; to no longer want to see patients die from the virus; they [healthcare professionals] must go ahead, and contribute with their knowledge in the fight against this virus; in every fight, there is a new story to learn” (Nurse 4, TN).

“[Relating] helped me to make an approximation of the day-to-day work; understand that, like patients, we suffer when things do not go as expected. That we are not half-gods, that every day we give all our effort to help the sick ones, to alleviate their suffering” (Doctor 11, TN).

“Healthcare professionals also have needs and feelings. However, being in the field and although it is not visible after the mask, we wear a big smile to be able to face a new day. Every day we convince ourselves that everything will work out and that we are going to overcome it, as we have done on other occasions. Throughout the day, bad news arrives from patients or colleagues and, gradually, you lose energy. A feeling of anger takes hold of you when you try to make it disappear as soon as possible, so as not to get home emotionally upset. This experience helps me to continue fighting to put ethical and moral principles before particular benefits. Furthermore, in times of the pandemic, where healthcare costs were magnified. Being better is of great hope when we shall defeat COVID-19” (Nurse 53, TN).

By reviewing the daily practices in the field of healthcare, the study participants were able to take stock of those situations that generated tension and that became an opportunity to think about subjectivity, as mentioned in these testimonies: “He taught me that you have to give value to all things and that you have to value the things of the others; It should also be in my professional practice: all people have value and contribute something good” (Nurse 44, S).

“Understand that a human being's life is very important; we learn daily to tackle our fears and to overcome them. This experience has been a stage of risks; we have learned to accept the SARS-COV2 virus as something normal in our daily lives” (Nurse 49, TN).

“It touches the depths of being, being able to know facts that have marked other people's lives; being able to have the opportunity to listen, learn, and handle different situations. This is what daily living is about and knowing how to cope with every opportunity” (Dentist 22, S).

“Every day we learn something different for our professional growth and as human beings” (Physiotherapist 3, S).

The ethics of care (Ricoeur, 1995, 2001) emphasizes the possibility of examining one's actions, for individual and collective growth, regarding moral and deontological tensions. These moral issues were relevant to this group of professionals: “sometimes we lose sight of our priorities because we are in charge of solving what it is urgent in labor matters; this activity helps to establish priorities and remember what is valuable” (Odontologist 3, S). Taking care of oneself involves valuing people and looking for pathways to configure one's subjectivity: “the importance of this story is the ability to establish or preserve moral integrity and to recover from distressing situations for ethical reasons” (Psychologist 1, TN). The ethical issue is present in many testimonies: “it invites us to reflect and become aware of that, if I do things well, without harming the other, I build family, business and society” (Nurse 43, S). Other participants mention similar issues:

“Knowing and exploring more as a person, avoiding limiting ourselves, to become mechanical. We play a professional role and the important thing is to contribute to societal growth; the important thing is to reflect before giving a preliminary judgment. Ethics and morals allow us to make decisions by reality, without resulting in a conflict, [how] we can be fair and good, based on the values and principles that we have.” (Nurse 45, S).

“It showed me the personal sensitivity that I can have and that it is important to put others first, ensure their well-being and, in the event of having staff under my responsibility in the future, I will know how to act” (Surgical Instrumentalist 1, S).

As already mentioned, the ethics of care also implies taking care of others to create a common shared concept of the “we” (Ricoeur, 1990; Gadamer, 2002). The different activities carried out in academic settings enable this: “It helped me understand the perspective of the lives of others” (Odontologist 6, S). There is the recognition of the other as a co-worker and professional in the field of healthcare, as it emerges in this testimony. The exercise makes it possible to “recognize the importance of others, their experiences and their knowledge […]. it helps me better understand the environment and look for tools to more effectively develop communication and work with others” (Doctor 27, S). It also enabled participants to accept “the value of the other; that has allowed me to assess the dimensions that the human being possesses. This activity can be extrapolated to the professional part, where we must interact with many people who are very valuable for themselves” (Medic 16, S).

The recognition of the patient, as the one who questions the exercise of other healthcare professionals and reflects upon the work that is carried out, was discussed in the following testimonials:

“It is difficult to see how the same teamwork stigmatizes patients, without addressing the reality of each person in depth; perhaps it is difficult to understand a situation until you are literally in the shoes of those people. As professionals, we have to provide holistic care to people, a space to vent and express all their feelings, providing them with security” (Nurse 12, TN).

One response outlined how “We learn that the care of our patients is done in a humanized way, with medical honesty, responsibility, and patient's freedom” (Nurse 37, RTN). As another participant outlined, “all human beings are susceptible to something and caretaking depends on us. It is also important to highlight those services; both administrative and healthcare ones must be streamlined and improved for better-humanized healthcare” (Physician 31, TN). This was reiterated by another response, outlining that “standing up for the rights of each patient so that they are not violated; the exercise of our profession honestly and transparently. Not to forget that healthcare careers are at the service of the community and are not only for profit” (Odontologist 3, TN).

These previous testimonies show the importance of the constitution of their subjectivity in intersubjective tension (Gadamer, 1975; Frank, 1995; ŽiŽek, 1999; Santos, 2010). The constitution of subjectivity, consequently, is embodied, insofar as the gaze is focused on memory and everyday life. On the other hand, the care of oneself and others are aspects that generate subjective well-being:

“It is liberating to relate the experience. It can be part of the process of healing emotions that is experienced with these types of moments where the fact of being a healthcare professional is so involved and, at the same time, being a relative of the one who lives the difficult situation” (Nurse 1, TN).

The participants recognized that these pedagogical settings made possible the conditions to constitute subjective well-being in the framework of the COVID-19 pandemic. They stated that elaborating narrative testimonies and working on valuable objects made it possible to reflect on themselves and meet others (Gadamer, 1986; Ricoeur, 1990). Thus, they positively valued the generation of the subjective well-being that the pedagogical actions developed:

“It was a wonderful interaction where relevant aspects were shared about the role we play in society, as individuals and as healthcare professionals” (Doctor 10, S). “It generated interaction with my colleagues, appreciating the positive things that other people have, highlighting their value and recognizing the positive in others” (Nurse 40, S). “We were able to express ourselves with affection and respect to each one; this makes us more aware of the situations of others” (Odontologist 13, S).

Within this framework, the pedagogical spaces that were promoted, offer opportunities for subjective well-being in postgraduate students in the framework of remote emergency education caused by the COVID-19 pandemic. This forum led this group of professionals to realize and reflect on ethics, and the situations that arise in day-to-day work, seek solutions, develop creativity, and provide a quality service.

The constitution of subjectivity and the ethics of care can also be understood as the space of interaction with the other, the place of the deployment of all potentialities, job satisfaction, appreciation, recognition, and closeness to the reality of others. Thus, subjective well-being was generated, as recognized by all participants.

## Results

Based on the discussions in the previous sections of this study, and taking into account narratives on the subjective well-being of this healthcare professional group in Colombia, this study contributes to understanding of subjective well-being.

As outlined in Table 1, a set of quantitative results shows the levels of acceptance of the activities carried out in the promotion of subjective well-being and the relevance of the actions carried out. When carrying out an analysis of the most frequently used words in the survey, certain concepts were raised, which allow us to compare the narrative findings with the results indicated in Table 1.

Displaying the triangulation with qualitative data, the participants' commitment to understanding subjective well-being in the eudaimonic horizon led to the establishment of the categories “Constitution of subjectivity” and “Ethics of care.” In the first category, memory and the everyday must be considered axial notions. In the second category, it is vital to consider caring for oneself and caring for others.

Such categories emerged from the narrative analysis and were also evident in the results of the reconnaissance survey. These findings showed that 98.78% of the participants recognized the importance of the activities carried out as a factor that generates subjective well-being, enabling them to recognize themselves as human beings and professionals. It is noteworthy that 99.39% of the participants considered the actions to be relevant, especially in the context of the COVID-19 pandemic.

The understanding of subjective wellbeing in the eudemonic horizon supports the categories “Constitution of subjectivity” and “Ethics of care.” In the first category, memory and the everyday must be considered as axial notions. In the second category, it is vital to consider caring for oneself and caring for others.

This study used memory, as a generator of subjective well-being, to frame situations, feelings, and significant events, generate approaches to affective situations, and cognitive approaches. First, it enabled participants to refer to emotions, frustrations, helplessness, and satisfaction with good work. Second, it facilitated reflections on acquired knowledge and practice in daily life, ethics, and morals in professional performance. An interesting indicator from the survey is the frequency of words such as *life (32), knowing (23) person (26)*, and *being (2*6), which refer to a recognition of the biographical trajectories of the participants, as outlined in Table 1.

Memory contributes to the expectations of the present and the future, based on lived experiences. All events have internal implications in people, as they imply a change in attitudes, appreciation of family, and the profession itself. Remembering enables a person to make decisions and value the others. Looking at the everyday enables people to review what frames subjective well-being, and in this case, professionals who work in healthcare. This was evidenced in the narratives of participants, 98.78% of whom considered that activities with valuable objects (photographs) generated self-knowledge. This was also seen in the frequency of words that appeared in the survey, for example *things (23), professional (22)*, and *objects (17*), which refer to the perception of the everyday as a primary category (see Table 1). Thus, the everyday becomes an opportunity to interact with others and satisfy the need to belong and relate socially.

The present study has shown that promoting memory activities in everyday life as categories of the constitution of subjectivity, provides the opportunity to reflect on the decision-making connected with ethical experience as part of the wider search for good and happiness. Such a situation becomes a challenge for education, from the perspective of subjective well-being.

Taking care of oneself and the promotion of subjective well-being in the academic space should be a constant practice, as it implies recognition and appreciation of oneself. Different words that appeared in the analysis of the rapid reconnaissance survey allowed participants to look at self-care. As outlined in Table 1, these words included *valuable (25), values, (22) knowledge, (21) ethics, (18) well-being (13)*, and *life (12)*. These words indicate that that self-care involves knowing oneself as an axiom. It involves reflecting on oneself, with an attitude of humility and truth, to realize what is happening inside ourselves and requires respect and consideration; forgiveness and acceptance of fears and failures, and, the “healing” of mistakes.

These aspects, which appeared emphatically in the narrative testimonies, were also evidenced in the reconnaissance survey when 98.78% of participants stated that the activities generated some type of well-being, as they had emerged in the narrative discourses on self-care and caring for the others compared to a 1.22%, who did not consider it this way. From this perspective, care also appears when 98.17% of students considered that the activities allowed them to meet their classmates.

Promoting self-care invites one to preserve one's health and seek a better quality of life, which provides a sense of well-being and personal satisfaction. Thus, subjective well-being is produced through self-care that guarantees physical and psychological well-being. In addition, reviewing how one acts or reacts facilitates self-evaluation and decision-making regarding behaviors and procedures.

Caring for others appears because caring for oneself is made explicit by action, through attitudes and behaviors that involve attention, knowledge, and techniques, to meet the needs of others. As shown in the narratives, as well in most used words in the rapid reconnaissance survey, the word *colleagues (18)* reflects the self-awareness about the others. This also happens for the word *ours (17)*, which allows thinking about common feelings and sensations (see Table 1).

For 99.39% of the participants, academic spaces acted as places of pedagogical experience that contributed to the training. In this perspective, and as it emerged in the narratives, the didactic interactions that were put into play derived in concrete moments of recognition of others, through the activities launched in this remote learning course mediated by technologies. In this respect, it is important to mention the frequency of the words *class (54), dynamics (17), knowledge (17) learning (10)*, and *tools (10), which* allowed us to recognize the didactic strength of the academic year. In the same way, the words: *thank you (37), excellent (21*), and *teacher (21)*, when contrasted with the narratives, made it possible to recognize the didactic dimensions in the promotion of subjective well-being. Therefore, it is feasible to promote subjective well-being actions in remote learning courses, emphasizing the constitution of subjectivity and self-care, as educational training opportunities.

In the academic spaces where these subjective well-being actions were carried out, contextualized learning was promoted from the perspective of practical knowledge, especially because the activities made ethical actions possible at an experiential narrative level, which facilitated the constitution of the subjectivity of the participants. These methods thus helped participants to recognize the importance of others, their experiences and knowledge, put themselves in the place of the other, and learn from the experiences of others. Participants placed themselves in different circumstances and learned from each one of them.

## Data Availability Statement

The datasets presented in this study can be found in online repositories. The names of the repository/repositories and accession number(s) can be found below: https://unisalleedu-my.sharepoint.com/:f:/g/personal/dibarragan_unisalle_edu_co/ErtJZ1kMKtJFg0uf3Xn9y48ByueSRGaoQNiX_7m-BeY0tA?e=94k3Bb.

## Ethics Statement

Ethical review and approval was not required for the study on human participants in accordance with the local legislation and institutional requirements. The patients/participants provided their written informed consent to participate in this study. Written informed consent was obtained from the individual(s) for the publication of any potentially identifiable images or data included in this article.

## Author Contributions

All authors listed have made a substantial, direct and intellectual contribution to the work, and approved it for publication.

## Conflict of Interest

The authors declare that the research was conducted in the absence of any commercial or financial relationships that could be construed as a potential conflict of interest.

## Publisher's Note

All claims expressed in this article are solely those of the authors and do not necessarily represent those of their affiliated organizations, or those of the publisher, the editors and the reviewers. Any product that may be evaluated in this article, or claim that may be made by its manufacturer, is not guaranteed or endorsed by the publisher.
